# Primary omental Gastrointestinal stromal tumor (GIST)

**DOI:** 10.1186/1477-7819-5-66

**Published:** 2007-06-12

**Authors:** Takeshi Todoroki, Takaaki Sano, Shinji Sakurai, Atsuki Segawa, Tamotsu Saitoh, Koichi Fujikawa, Shuji Yamada, Nobutsune Hirahara, Yoshito Tsushima, Ryuji Motojima, Teiji Motojima

**Affiliations:** 1 Department of Surgery, Motojima General Hospital, Ota, 373-0033 Japan; 2 Department of Tumor Pathology Gunma University, Maebashi, Japan; 3 Department of Gastroenterology, Motojima General Hospital, Ota, 373-0033 Japan; 4 Diagnostic Radiology and Nuclear Medicine, Gunma University, Maebashi, Japan

## Abstract

**Background:**

We report herein a rare case of primary omental gastrointestinal stromal tumor (GIST).

**Case presentation:**

A 65 year-old man was referred to our hospital with a huge abdominal mass occupying the entire left upper abdomen as shown by sonography. On computed tomography (CT), this appeared as a heterogeneous low-density mass with faint enhancement. Abdominal angiography revealed that the right gastroepiploic artery supplied the tumor. With such an indication of gastric GIST, liposarcoma, leiomyosarcoma or mesothelioma laparotomy was performed and revealed that this large mass measured 20 × 17 × 6 cm, arising from the greater omentum. It was completely resected. Histopathologically, it was composed of proliferating spindle and epithelioid cells with an interlacing bundle pattern. Immunohistochemically, the tumor was positive for myeloid stem cell antigen (CD34), weakly positive for c-KIT (CD117) and slightly positive for neuron-specific enolase (NSE), but negative for cytokeratin (CK), alpha-smooth muscle actin (SMA) and S-100 protein. A mutation was identified in the platelet-derived growth factor alpha (PDGFRA) juxtamembrane domain (exon 12, codon561) and the tumor was diagnosed as an omental GIST. The postoperative course was uneventful. The patient is treated by Glevec^® ^and is alive well with no sign of relapse.

**Conclusion:**

Our case demonstrated a weak immunohistochemical expression of c-kit (CD117) and a point mutation in PDGFRA exon 12 resulting in an Asp for Val^561 ^substitution. Imatinib therapy as an adjuvant to complete resection has been carried out safely. Because of the rarity of primary omental GISTs, it is inevitable to analyze accumulating data from case reports for a better and more detailed understanding of primary omental GISTs.

## Background

GISTs are mesenchymal tumors, the majority of which is KIT (CD117)-positive, typically arising in association with the muscularis propria of the gastrointestinal tract wall. Accordingly, they occur most frequently in the stomach (60%), jejunum and ileum (30%), and less frequently in the duodenum (5%), < 5% colorectal < 1% in the esophagus and appendix [1]. A small number may originate not from the omentum, but from outside the gastrointestinal tract; these are designated extra-GISTs (EGISTs)[2]. To the best of our knowledge, thus far only 28 cases of primary omental GISTs, including that described in the present study, have been reported. Histopathologic and immunohistochemical characteristics of EGISTs are similar to GISTs, the majority of both having mutually exclusive gain-of-function KIT/PDGFRA mutations. However, because of the rarity of the omental GISTs, it is difficult to assess their malignant potential, prognostic factors or efficacy of surgery alone or in combination with molecular targeting chemotherapy with the Kit/PDGFRA tyrosine kinase inhibitor Imatinib, and their overall prognosis.

Here, we report a rare case of primary omental myxoid epithelioid GIST which we have characterized immunohistochemically and genetically. We review the English-language literature on primary omental GISTs.

## Case presentation

**Figure 1:**
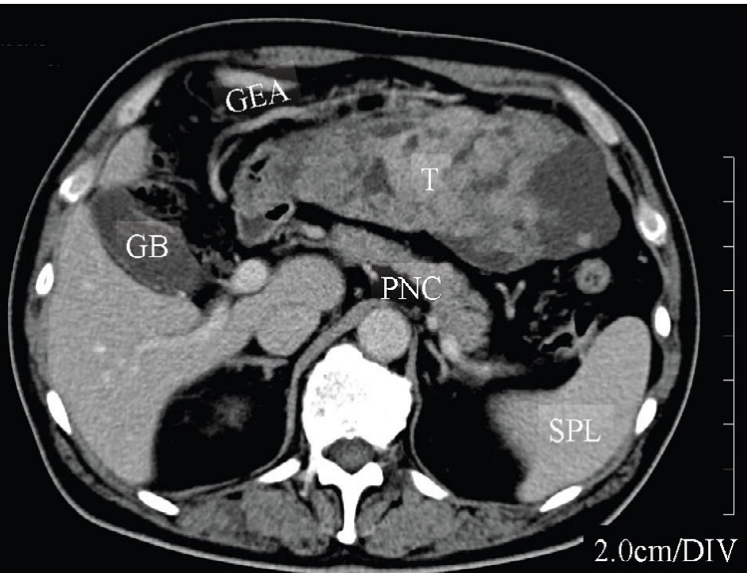
**Computed Tomography**. A huge tumor behind the left hepatic lobe showed heterogeneous low density with faint enhancement. GB, gallbladder; GEA, gastroepiploic artery; T, tumor; PNC, pancreas; SPL, spleen.

**Figure 2:**
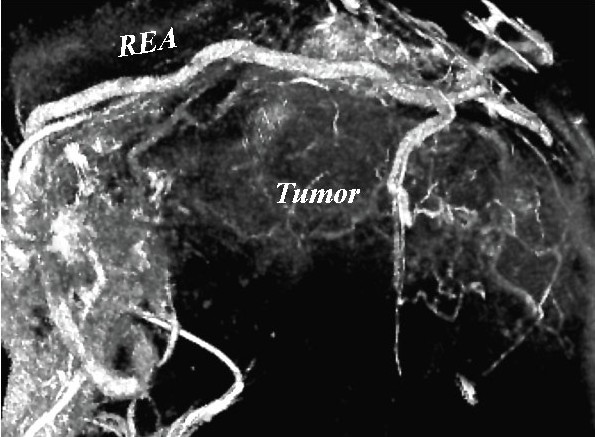
**3-D reconstructed angiography of the gastroepiploic artery**. A major tumor-supply artery diverges from the right gastroepiploic artery (REA).

**Figure 3:**
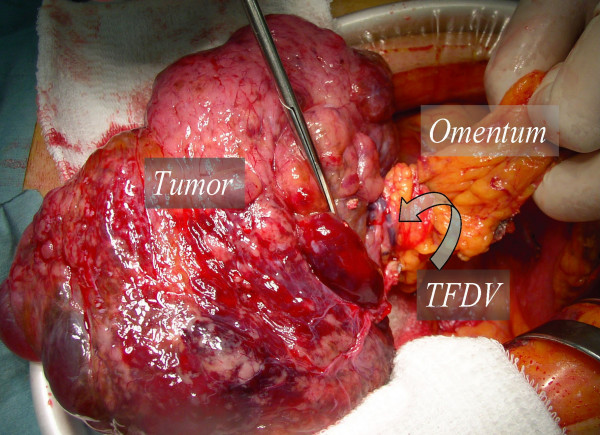
**Intra-operative photograph**. Scissors indicate major tumor-feeding vessels (TFDV).

**Figure 4:**
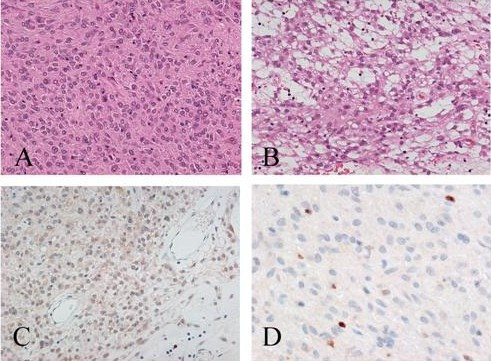
**Photomicrographs of the primary omental GIST tumor**. A. Epithelioid components of GIST. Tumor cells show eosinophilic cytoplasm and peripherally placed nuclei, and are mostly cohesive (H&E). B. Some components show a spindle cell pattern with myxoid stroma (H&E). C. Tumor cells weakly immunoreactive for c-kit (CD117). D. Immunostaining for MIB-1: sparse of positive tumor cells are shown (MIB-1 index: 4.4%).

**Figure 5:**
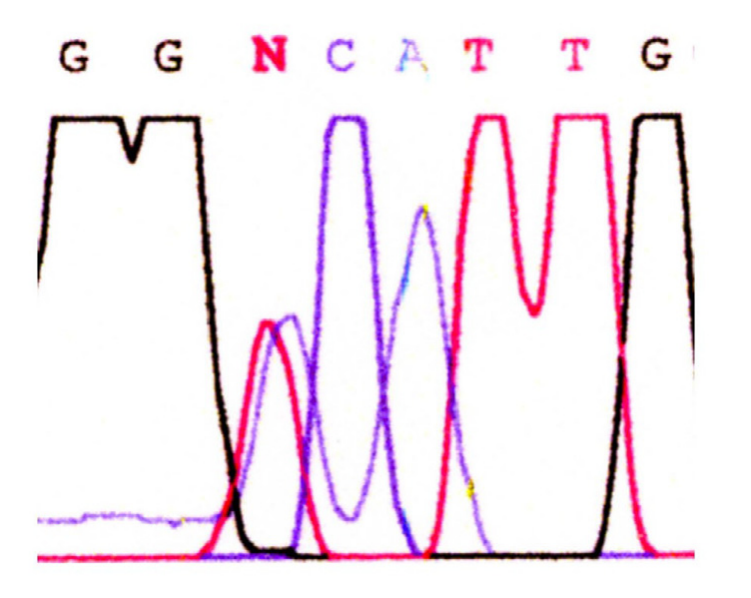
**Genomic sequencing of the PDGFRA gene**. Direct sequencing analysis showing a point mutation at codon 561 (GTC to GAC) in exon12. Val561 is changed to Asp.

A 65-year-old man was referred to our hospital in October 2006 with a huge abdominal tumor. A firm mass was palpated extending from the epigastrium to the left hypogastrium. There were no laboratory abnormalities, except a slight elevation of the total bilirubin (1.3 mg/dl) and lactate dehydrogenase (LDH: 217 IU/L) levels in the serum. The tumor markers carcinoembryonic antigen (CEA) and carbohydrate antigen (CA19-9) were within the normal range. Ultrasonography showed that the mass occupied almost the entire upper abdomen anterior to the bowel loops. On computed tomography (CT), a mass behind the left hepatic lobe showed heterogeneous low density with faint enhancement (Fig. 1). Abdominal angiography revealed that the tumor was vascularized mainly from the right epigastric artery (Fig. 2). We suspected liposarcoma, leiomyosarcoma, mesothelioma, or gastric GIST. At laparotomy on October 2006, a well-encapsulated tumor was found in the greater omentum. There was no adhesion to adjacent organs and structures but a pinpoint adhesion to the stomach. The right gastroepiploic artery and vein were prominent and stretched by the tumor, and a major supply vessel diverged from it in one stalk (Fig. 3). There was no evidence of metastasis in the abdominal cavity. Grossly, a well-demarcated reddish-gray solid tumor, 20 × 17 × 6 cm in size, showed irregular modularity (Fig 3). The cut surfaces were tan-colored and contained focally necrotic areas and a cystic nodule. Histopathologically, the tumor was composed of proliferating epithelioid cells and myxoid cells with an interlacing bundle pattern (Fig. 4A &4B). The cellularity was relatively high and the frequency of mitotic figures was 2 of 50 high power fields (HPF). The MIB-1 index was 4.4% (Fig. 4D). The tumor cells were diffusely immunoreactive for myeloid stem cell antigen (CD34), weakly or focally positive for c-kit proto-oncogene protein product (CD117) (Fig. 4C) and slightly positive for neuron-specific enolase (NSE). However, there was no staining for cytokeratin (CK), alpha-smooth muscle actin (SMA) or S-100 protein. Direct sequencing demonstrated mutations in the platelet-derived growth factor alpha (PDGFRA) gene exon 12, codon 561, encoding a thymine to adenine substitution (Fig. 5). These findings were consistent with a myxoid epithelioid GIST lacking myogenic features and neural attributes. The patient had a complete tumor resection and an uneventful postoperative course. He was treated by per os administration of Glevec^® ^300 mg/day as an adjuvant postoperative molecular targeting chemotherapy and has been living disease-free for 6 months.

## Discussion

GISTs are mesenchymal tumors originating primarily from interstitial cells of Cajal or related stem cell-like precursors [3] of the gastrointestinal tract wall. Typically, they are characterized by the expression of the receptor tyrosine kinase Kit (CD117)[4], although some GISTs do not or only weakly express this marker [5]. Primary omental GISTs, Sakurai et al. implicated their possible pathogenesis from ICC-like Kit-positive cells existing in the normal omentum [6]. It is commonly accepted that mutually exclusive mutations in Kit or PDGFRA receptor tyrosine kinase proteins play a central role in GIST pathogenesis [7-10]. This is clearly illustrated by the collected data from primary omental GISTs (Table 2). Mutually exclusive gain-of-function Kit or PDGFRA mutations represent in-frame deletions, point mutations, duplications or insertions. Mutations in the Kit juxtamembrane domain (exon 11) are the most common in GISTs of all sites, whereas a rare Kit extracellular domain (exon 9) Ala502-Tyr503 duplication is specific for intestinal GISTs. Mutations in PDGFRA have been identified in the juxtamembrane (exon 12), as observed in our case, and tyrosine kinase domains (exons 14 and 18), nearly exclusively in gastric GISTs, mostly epithelioid variants. Some Kit and PDGFRA mutations carry prognostic value. The Kit/PDGFRA tyrosine kinase inhibitor Imatinib has been successfully used in the treatment of metastatic GISTs for more than 5 years [10,11]. Microscopic features are site-dependent and the majority (more than 85%) appears as spindle cell tumors [11,12]. However, only half of our collected omental GISTs appeared to be spindle cell tumors, the remaining being epithelioid and myxoid (Table 1). No correlation between prognosis and histologic type has been reported. On the other hand, it is well known that tumor size and mitotic activity are the best prognostic features [10]. In our case, the tumor size and the mitotic activity expressed per 50 HPFs were 20 cm and 2, respectively. It is hardly predict accurately the risk for disease progression and malignant potential of our case, because of scarcity of the primary omental GIST. Based on the criteria advocated by Miettinen and Lasota, these two tumor parameters place the tumor in group 3b, at the intermediate risk [10], although according to the risk assessment proposed by Fletcher, et al., our patient displayed high-risk feature (tumor size above 10 cm, irrespective of low number of mitoses) [13].

**Table 1:** Case review of the primary omental GISTs

**Case No.**	**Authors (Year)**	**Age/Gender**	**Size (cm)**	**Histology**	**Mitosis/50HPF**	**Outcome**
1	Takahashi [17] (1998)	71/M	17	Sp	1–3	ANED, 2-Mo
2	Miettinen [18] (1999)	58/F	2.5	Ep	1	Dead of colon cancer, 2.3-Yrs
3		89/M	2.5	Mixed	7	DUC, 3-Yrs
4		31/F	7.5	Sp	19	ANED, 3.5-Yrs
5		80/F	10	Ep	7	ANED, 2.0-Yrs
6		44/M	12	Ep	<1	ANED, 1.6-Yrs
7		72/M	15	Mixed	26	ANED, 1.5-Yrs
8		67/F	16.5	Sp	5	LTF
9		56/F	20	Ep	0	LTF
10		64/M	20	Sp	4	ANED, 2.0-Yrs
11		34/M	23	Sp	1	LTF
12		60/M	24	Ep	1	ANED, 3.4-Yrs
13		68/F	26	Sp	2	ANED, 8.5-Yrs
14		70/F	36	Ep	<1	LTF
15	Sakurai [6] (2001)	39/F	6	Sp	7.7**	NA
16		52/F	11.5	Sp	4.3**	NA
17		74/F	8	Sp	<1**	NA
18		65/F	16	Sp	0.9**	NA
19		61/F	23	Sp	22**	NA
20	Fukuda [19] (2001)	45/M	4.5	Sp	<1*	ANED, 0.9-Yrs
21	Suzuki [15] (2003)	65/M	13	Sp	5–8 13.8*	DOD 1.3-Yrs (Liver mets.)
22	Sakurai [8] (2004)	73/F	4	Myxoid	3*	ANED, 4-Mo
23		52/M	>20	Ep	4*	ANED, 13-Mo
24	Yamamoto [9](2004)	62/F	11	Ep	3	ANED, 0.5-Yrs
25		54//M	15	Ep	3	ANED, 5.2-Yrs
26		49/F	17	Ep	1	ANED, 4.0-Yrs
27	Caricato [20] (2005)	84/F	≤5	Sp	NA	ANED, 0.3-Yrs
28	Present case (2006)	65/M	20	Myxoid	2	ANED, 0.5-Yrs

Ep: epithelioid, SP: spindle, ANED: alive with no evident disease, Mo: month, Yrs: years, DUC: Dead of unknown cause, *: MIB-1-labeling index. **: K-67 labeling index (%), LFT: lost to follow-up, NA: not available, DOD: dead of disease,

**Table 2:** Immunohistochemistry and Mutations in the primary omental GISTs

Case No.	Histology	Immunohistochemistry					Mutations	
		KIT	CD34	S100	SMA	Desmin	Gene	Site

15	Spindle	Positive	+	-	-	-	C-kit	Exon11, MS
16	Spindle	Positive	+	-	-	-	C-kit	Exon11, IFD
17	Spindle	Positive	+	-	-	-	C-kit	Exon11, MS
18	Spindle	Positive	+	-	-	-	C-kit	Exon11, MS
19	Spindle	Positive	+	-	Weak	-	C-kit	Exon11, IFD
22	Myxoid epithelioid	Weak	+	-	+	-	PDGFRA	Exon18 del D842V
23	Epithelioid	Weak	-	-	-	-	PDGFRA	Exon18 DIMH842-845
26	Epithelioid	Weak	+	NA	NA	NA	PDGFRA	Exon18
28	Myxoid epithelioid	Weak	+	-	-	-	PDGFRA	Exon12, V561D

MS: miss sense, IFD: in-frame deletion, NA: not available

Because of possible relapse even after complete resection of omental GISTs [14-16] and the objective response rate of 67 % of Imatinib to the mutation in PDGFRA exon 12 [11], our patient received daily oral administration of 300 mg Glevec^®^, (we applied 15% reduced dose, referring to a report by Cormier, et al. after took account of a smaller average of Japanese build than American) [14,16]. At the present time, there are no signs of toxicity and no evidence of relapse. However, because of the short follow-up period and rarity of the primary omental GISTs, it is difficult to assess appropriately their malignant potential, efficacy of different treatment procedures and their overall prognosis. In order to improve overall understanding of the primary omental GISTs, it is useful to analyze the collected detailed data from reported cases.

## Conclusion

Our case demonstrated a weak immunohistochemical expression of c-kit (CD117) and a point mutation in PDGFRA exon 12 resulting in an Asp for Val^561 ^substitution. Because of the rarity of primary omental GISTs, it is difficult to assess their malignant potential and their overall prognosis. Imatinib therapy as an adjuvant to complete resection has been carried out safely and may prevent relapse to prolong long-term survival. It is essential to analyze accumulating data from case reports for a better and more detailed understanding of primary omental GISTs.

## Competing interests

The author(s) declare that they have no competing interests.

## Authors' contributions

TS, SS and AS had contributed as molecular tumor pathologist. TS, KF, SY, RM and TM contributed as a gastrointestinal surgeon in operative performance and postoperative treatment. NH and YT contributed as gastroenterologist and diagnostic radiologist.

All authors read and approved the final manuscript.
